# Cardiac Biomarkers

**DOI:** 10.1155/2015/370569

**Published:** 2015-04-19

**Authors:** Johannes Mair, Allan Jaffe, Fred Apple, Bertil Lindahl

**Affiliations:** ^1^Department of Internal Medicine III (Cardiology and Angiology), Innsbruck Medical University, 6020 Innsbruck, Austria; ^2^Division of Cardiovascular Diseases and Internal Medicine, Mayo Clinic and Medical School, Rochester, MN 55905, USA; ^3^Department of Laboratory Medicine and Pathology, Hennepin County Medical Center and University of Minnesota School of Medicine, Minneapolis, MN 55415, USA; ^4^Department of Medical Sciences, Uppsala Clinical Research Center, Uppsala University, 75237 Uppsala, Sweden

Cardiac biomarkers have evolved as essential tools in cardiology over the last 50 years, that is, for primary and secondary prevention, the diagnosis and management of acute myocardial infarction (AMI), and the diagnosis and risk stratification of heart failure (HF). We are beginning an era when it may be possible for biomarkers to direct treatment to optimize patient management. This is already the case with cardiac troponin (cTn) but should be the goal with all biomarkers. This special issue is a compilation of timely reviews and original articles on this topic.

More than 60 years ago in 1954 Karmen et al. [1] first reported that release of aspartate aminotransferase (AST), formerly glutamate oxaloacetate transaminase (GOT), from necrotic cardiac myocytes could be detected in the serum and could aid in the diagnosis of AMI. This initiated the era of enzymology in cardiology. In 1955 lactate dehydrogenase (LDH) was first published as a marker of AMI [2], and a direct enzymatic assay for *α*-hydroxybutyrate dehydrogenase activity was later developed to increase cardiac specificity [3]. Subsequently an effective enzymatic assay for the quantification of creatine kinase (CK) activity was developed by Rosalki [4] and established this enzyme as the standard marker for the detection of muscle damage. CK remained the mainstay for AMI diagnosis for about 20 years. The development of immunoinhibition assays [5] for activity measurement of the more cardiac-specific isoenzyme CKMB on automated analyzers made this test and marker popular and widely used for many years. During the same period the Framingham study [6] established lipid concentrations and in particular cholesterol measured by enzymatic assays as risk factors for the development of cardiovascular diseases.

In the 1970s radioimmunoassays were developed and revolutionized laboratory medicine including AMI diagnosis. Immunoassays for myoglobin and CKMB were developed [7, 8]. Subsequently immunoassays were substantially improved by using monoclonal antibodies, and rapid immunoassays for measuring the so-called CKMB “mass” replaced CKMB activity measurements as the criterion standard for AMI diagnosis. Given the limitations of CKMB regarding cardiac specificity there was an intense search for potentially more specific cardiac damage markers in the 1980s. Finally only cardiac troponin I (cTnI) and cardiac troponin T (cTnT) turned out to be truly cardiac-specific and made the way from research to clinical routine use [9, 10]. This initiated the troponin era in the laboratory diagnosis of myocardial injury. Major interest was generated by studies indicating that cardiac troponins were useful for risk stratification and in identifying individuals most apt to benefit from an early invasive strategy in patients with acute coronary syndromes (ACS) [11–14], and a new key role for cardiac biomarkers was added to the traditional diagnostic role. Subsequent large clinical studies confirmed this prognostic role convincingly for both cTnT and cTnI, and also because of their additional outstanding cardiac specificity they were proposed as the new golden standard for the laboratory diagnosis of myocardial injury in a consensus statement published in 2000 [13]. cTnI and cTnT have remained criterion laboratory markers for the diagnosis of myocardial injury since then including in the subsequently published Universal Definitions of AMI [14]. During recent years the analytical sensitivities of cTnI and cTnT assays have improved remarkably to a degree that could not be expected at the beginning of the “immunoassay era,” and recently, the so-called “high-sensitivity” cTnT and cTnI assays have been introduced in routine clinical practice. These assays enable earlier detection of AMI obviating the need for other “early” necrosis markers, and with these assays cTn can be detected even in the majority of normal individuals [15].

About the same time in 1981, de Bold et al. made the observation that atrial myocardial extracts, when injected in rats, resulted in rapid and important natriuretic response [16]. This finally led to the discovery of the cardiac natriuretic peptide system. Natriuretic peptides are primarily synthesized in the heart and upregulated by myocardial stress mediated by volume, or pressure overload. B-type natriuretic peptide (BNP) and the N-terminal split product of its precursor hormone proBNP as well as N-terminal proatrial natriuretic peptide (ANP) turned out to be suitable laboratory markers for routine diagnosis and risk stratification of HF which opened totally new applications of laboratory testing in cardiology [17].

The 1990s was the golden era of cardiac biomarkers. The great clinical significance and economic impact of cardiac diseases triggered a huge research effort in the discovery of novel biomarkers for the diagnosis and risk stratification of ACS and HF as well as risk stratification in primary and secondary prevention. This led to the discovery of numerous biomarkers and the development of immunoassays which were also suitable for routine measurement. The main focus was on markers of coronary plaque formation, plaque destabilization, intracoronary thrombus formation (coagulation and platelet activation, reduced endogenous fibrinolytic activity), and markers of myocardial ischemia. However, the vast majority of these markers did not make the way from research to routine application due to analytical issues or because the clinical impact for risk stratification was limited because they did not add much to traditional risk factors and even in multimarker approach improved risk stratification and patient reclassification only very modestly. Critically, they did not lead to direct information about how to improve patient management.

During this period also genomic biomarkers entered the field and have been particularly popular in the last two decades. Almost all of the candidate-gene era genetic biomarkers of cardiovascular disease failed to be validated after an initial period of enthusiasm [18]. Rare variants may be potent but because they are rare, they do not identify large numbers of additional patients at risk. Common variants such as single genetic variants confer extremely small risks such that the usual way of calculating risk, such as ascertaining smoking habits and measuring blood pressure and cholesterol, is better than analyses for these commonly occurring variations in DNA sequences. Consequently, the current consensus is not to test for commonly occurring genetic variants with weak effects [19]. Currently, the study of microRNAs has also become a very popular area of research. MicroRNAs are small, regulatory, usually inhibitory noncoding RNAs which can be also detected in blood and could serve as biomarkers in cardiovascular disease [20]. However, it remains to be shown whether microRNAs will be relevant for routine cardiovascular diagnosis, risk stratification, or direction of therapy.

A possibility to potentially overcome limitations of single biomarkers is to combine them in multimarker panel testing to strengthen their clinical utility by combining the information of different aspects of the pathophysiology of cardiac diseases. This multimarker approach has been extensively studied but a breakthrough in this area has not yet occurred [21]. Future approaches could also combine proteins, lipids, metabolites, genetic markers, and imaging technologies. It is likely that, over time, panels will emerge as valuable in this area.

In summary, the role of biomarkers in cardiovascular diseases, such as AMI and HF, is very well established with cardiac troponin and natriuretic peptide testing as essential parts of patient evaluation. Despite major efforts in recent years, biomarkers for the prediction of coronary artery disease (CAD) and for risk stratification in stable and unstable CAD or the general population have not yet fulfilled their manifest promise [21]. The most established marker in this respect is high-sensitivity C-reactive protein which still remains controversial. It will be difficult for new cardiac biomarkers to substantially add to the information which can be obtained from the results of high-sensitivity cardiac troponin and natriuretic peptides.

## References

[1] Karmen A., Wroblewski F., La Due J. S. (1955). Transaminase activity in human blood. *The Journal of Clinical Investigation*.

[2] Wroblewski F., la Due J. S. (1955). Lactic dehydrogenase activity in blood. *Proceedings of the Society for Experimental Biology and Medicine*.

[3] Rosalki S. B., Wilkinson J. H. (1960). Reduction of *α*-ketobutyrate by human serum. *Nature*.

[4] Rosalki S. B. (1967). An improved procedure for serum creatine phosphokinase determination. *The Journal of Laboratory and Clinical Medicine*.

[5] Jockers-Wretou E., Pfleiderer G. (1975). Quantitation of creatine kinase isoenzymes in human tissues and sera by an immunological method. *Clinica Chimica Acta*.

[6] Kannel W. B., Dawber T. R., Friedman G. D., Glennon W. E., McNamara P. M. (1964). Risk factors in coronary heart disease. An evaluation of several serum lipids as predictors of coronary heart disease; The Framingham study. *Annals of Internal Medicine*.

[7] Stone M. J., Willerson J. T., Gomez Sanchez C. E., Waterman M. R. (1975). Radioimmunoassay of myoglobin in human serum. Results in patients with acute myocardial infarction. *The Journal of Clinical Investigation*.

[8] Roberts R., Sobel B. E., Parker C. W. (1976). Radioimmunoassay for creatine kinase isoenzymes. *Science*.

[9] Cummins B., Auckland M. L., Cummins P. (1987). Cardiac-specific troponin-I radioimmunoassay in the diagnosis of acute myocardial infarction. *American Heart Journal*.

[10] Katus H. A., Remppis A., Looser S., Hallermeier K., Scheffold T., Kubler W. (1989). Enzyme linked immuno assay of cardiac troponin T for the detection of acute myocardial infarction in patients. *Journal of Molecular and Cellular Cardiology*.

[11] Hamm C. W., Ravkilde J., Gerhardt W. (1992). The prognostic value of serum troponin T in unstable angina. *The New England Journal of Medicine*.

[12] Morrow D. A., Cannon C. P., Rifai N. (2001). Ability of minor elevations of troponins I and T to predict benefit from an early invasive strategy in patients with unstable angina and non-ST elevation myocardial infarction: results from a randomized trial. *Journal of the American Medical Association*.

[13] Alpert J. S., Thygesen K., Antman E. (2000). Myocardial infarction redefined—a consensus document of the Joint European Society of Cardiology/American College of Cardiology Committee for the redefinition of myocardial infarction. *Journal of the American College of Cardiology*.

[14] Thygesen K., Alpert J. S., Jaffe A. S., Simoons M. L., Chaitman B. R., White H. D. (2012). Joint ESC/ACCF/AHA/WHF task force for the universal definition of myocardial infarction. Third universal definition of myocardial infarction. *European Heart Journal*.

[15] Thygesen K., Mair J., Giannitsis E. (2012). How to use high-sensitivity cardiac troponins in acute cardiac care. *European Heart Journal*.

[16] de Bold A. J., Borenstein H. B., Veress A. T., Sonnenberg H. (1981). A rapid and potent natriuretic response to intravenous injection of atrial myocardial extract in rats. *Life Sciences*.

[17] Thygesen K., Mair J., Mueller C. (2012). Recommendations for the use of natriuretic peptides in acute cardiac care: a position statement from the Study Group on Biomarkers in Cardiology of the ESC Working Group on Acute Cardiac Care. *European Heart Journal*.

[18] Ntzani E. E., Rizos E. C., Ioannidis J. P. A. (2007). Genetic effects versus bias for candidate polymorphisms in myocardial infarction: case study and overview of large-scale evidence. *American Journal of Epidemiology*.

[19] Faergeman O. (2013). Genes and cardiovascular risk. *European Heart Journal*.

[20] Latronico M. V. G., Catalucci D., Condorelli G. (2007). Emerging role of microRNAs in cardiovascular biology (review). *Circulation Research*.

[21] Melander O., Newton-Cheh C., Almgren P. (2009). Novel and conventional biomarkers for prediction of incident cardiovascular events in the community. *The Journal of the American Medical Association*.

